# An Immunogram for an Individualized Assessment of the Antitumor Immune Response in Patients With Hepatocellular Carcinoma

**DOI:** 10.3389/fonc.2020.01189

**Published:** 2020-07-31

**Authors:** Ying Hu, Huaibo Sun, Henghui Zhang, Xianbo Wang

**Affiliations:** ^1^Center of Integrative Medicine, Beijing Ditan Hospital, Capital Medical University, Beijing, China; ^2^Genecast Precision Medicine Technology Institute, Genecast Biotechnology Co., Ltd., Beijing, China; ^3^Institute of Infectious Diseases, Beijing Ditan Hospital, Capital Medical University, Beijing, China

**Keywords:** hepatocellular carcinoma, immunogram, cancer-immunity cycle, antitumor immune response, prognosis

## Abstract

In clinical practice, the cancer-immunity cycle of an individual patient with hepatocellular carcinoma (HCC) must be described to support the clinical management of cancer. The present study explored the immunograms of patients with liver cancer based on liver RNA sequencing data to visually display the individualized cancer-immunity cycles. Two independent HCC cohorts [The Cancer Genome Atlas (TCGA) and Liver Cancer-RIKEN, Japan (LIRI-JP) HCC cohorts] with whole exome sequencing (WES) data, RNA sequencing data, and clinical data from TCGA and International Cancer Genome Consortium (ICGC) were enrolled in this study. This study constructed HCC immunograms of cancer immune cells to visually explore the anticancer immune responses of patients with HCC. The patterns of the HCC immunograms were categorized into two clusters: hot and cold HCC immunograms. Favorable overall survival (OS) and disease-free survival (DFS) were observed in the hot immunogram cluster in the TCGA cohort. The results for LIRI-JP cohort were similar to the TCGA cohort. The OS of patients with HCC presenting the hot immunogram was longer than patients with the cold immunogram in the LIRI-JP HCC cohort. Compared with cold immunograms, hot immunograms were characterized by higher levels of immune cell infiltration and stronger immune signatures, including cytolytic activity, IFN-γ signature, immunocostimulator, immunoinhibitor, chemokine, adhesion molecule, MHC I, MHC II, and non-class MHC levels. The main difference in molecular features between hot and cold immunograms was reflected in WNT-CTNNB1 alterations and copy number variant (CNV) and loss of heterozygosity (LOH) scores, which are the molecular features associated with resistance to immunotherapy and tumor escape. The immunogram patterns were distinct in terms of the different molecular features of HCC tumors. The HCC immunogram for the cancer-immune cycle was able to visualize the personalized antitumor immune response of patients with HCC, and the patterns of the HCC immunograms contributed to the clinical outcomes of patients, which may facilitate an individualized assessment of the antitumor immune response for optimal personalized immunotherapy.

## Introduction

An extensive clinical study has shown that cancer immunotherapy is a key component of the clinical management of cancer (1–3). A comprehensive understanding of the cancer–immune system interaction is crucial for developing new drugs and clinical strategies. Daniel S. Chen and Ira Mellman proposed the cancer-immunity cycle to illustrate the steps of the antitumor immune response, including the release of cancer cell antigens, cancer antigen presentation, priming, and activation, trafficking of T cells to tumors, infiltration of T cells into tumors, recognition of cancer cells by T cells, and killing of cancer cells, and to obtain a better understanding of the interactions between cancer and the immune system (4). From the perspective of the cancer-immunity cycles, cancer immunotherapy mainly includes two classes as described below. One class of immunotherapy is designed to improve the stimulatory immune factors, such as adoptive T cell therapy, which may lead the revolution of the cancer immunity cycle and potentially enhances the eventual self-propagation of the cycle (4). The other class of immunotherapy is designed to prevent immune effector inhibition, such as PD-1/PD-L1 blockade, which reinvigorates and potentially expands the pre-existing anticancer immune response (4, 5).

As described above, different immunotherapies are designed to regulate distinct dimensions of the cancer-immunity cycle. Therefore, an evaluation of the cancer-immunity cycle of individual patients is the basis for implementing clinical strategies tailored to each patient. Based on the theory of the cancer-immunity cycle, Jun Nakajima constructed an immunogram that visually illustrates the cancer-immunity cycle of individual patients with lung cancer (6). The immunogram for the cancer-immunity cycle integrated all exam and RNA sequencing data, followed by the cloud transformation of the complex omics data in a radar plot to display the immune response of each patient. The steps of the cancer-immunity cycle were assessed using eight axes of the immunogram score (IGS), which included T cell immunity (IGS1), tumor antigenicity (IGS2), priming and activation (IGS3), trafficking and infiltration (IGS4), recognition of tumor cells (IGS5), inhibitor cells (IGS6), checkpoint expression (IGS7), and inhibitory molecules (IGS8). The immunogram will assist clinicians in making personalized medical decisions for each patient.

The liver is an immunological organ that contains numerous adaptive and innate immune cells (7). The liver is also a special anatomical organ in which the antigen-rich blood is scanned by antigen-presenting cells and lymphocytes through a network of sinusoids (8). Additionally, major hepatocellular carcinoma (HCC) occurs in patients with underlying chronic liver inflammation associated with hepatitis B or C virus infections, alcohol abuse, and fatty liver (9). Therefore, the immune microenvironment plays a vital role in HCC development. Previous studies illustrated the immune landscape of liver cancer based on single-cell data, RNA sequencing data, and T cell receptor sequence data (10).

In clinical practice, the cancer-immunity cycle of individual patients with HCC must be described to support the clinical management of cancer. However, few studies have been published in this field. The present study explored immunograms of patients with HCC based on liver RNA-Seq data to visually display the personal cancer-immunity cycle. Moreover, we investigated the HCC immunogram and clinical outcomes and the correlation between the HCC immunogram and molecular features to better understand the individual anticancer immune response in the liver and support personal clinical decision making.

## Materials and Methods

### HCC Cohort and Data Collection and Preprocessing

**Figure 1:**
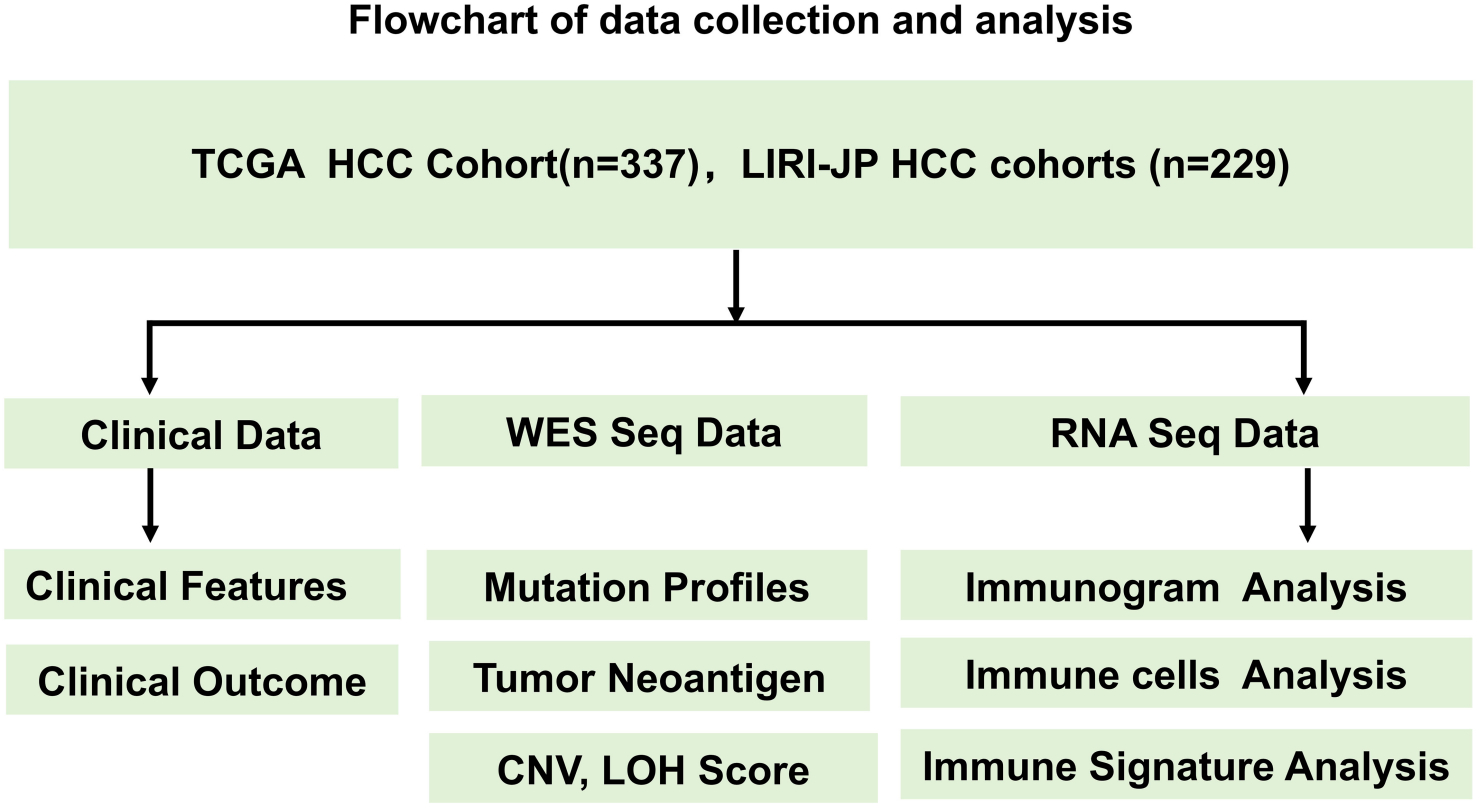
Flowchart of the data collection and analysis process. The design of the study and the process of data selection are shown. Two independent HCC cohorts (TCGA and LIRI-JP HCC cohorts) with WES data, RNA sequencing data, and clinical data from TCGA and ICGC were enrolled in this study.

The design of the study and the process of data selection are shown in Figure 1. We searched for HCC cohorts with whole exome sequencing (WES), RNA sequencing, and clinical data from The Cancer Genome Atlas (TCGA) and International Cancer Genome Consortium (ICGC). LIHC-TCGA and LIRI-JP HCC cohorts were enrolled in this study.

LIHC-TCGA was selected to explore the relations of the HCC immunogram and clinical outcomes, and LIRI-JP HCC was selected as an independent cohort to validate the prognostic value of the HCC immunogram. Data from the LIHC-TCGA and LIRI-JP HCC cohorts were downloaded from the UCSC Xena browser. The values of the RNA sequencing data (FPKM) were transformed into transcripts per million kilobase (TPM) values. The clinical information for the HCC cohort is shown in Table 1.

**Table 1:** HCC immunogram cluster and clinical features.

**Variable**	***N***	**Cold immunogram**	**Hot immunogram**	***P*-value**
Age				0.320
<60 years	156	94	62	
≥60 years	180	98	82	
Gender				0.411
Male	228	126	102	
Female	109	66	43	
Etiology				0.056
Hepatitis B	94	58	36	
Hepatitis C	45	20	25	
Hepatitis B and C	6	1	5	
NAFLD	15	8	7	
Alcohol consumption	66	34	32	
Others	111	71	40	
Vascular invasion				0.589
Microvascular infiltration	81	50	31	
Macrovascular infiltration	14	7	7	
None	188	105	83	
Unknown	54	30	24	
Fibrosis				0.334
No fibrosis	66	43	23	
Portal fibrosis	27	13	14	
Fibrous septa	27	18	9	
Nodular formation	6	3	3	
Established cirrhosis	65	36	29	
Unknown	146	79	67	
Stage				0.055
I	156	82	74	
II	77	41	36	
III	80	54	26	
IV	5	3	2	
Child-pugh classification grade				0.611
A	199	117	82	
B	19	13	6	
C	1	0	1	
Unknown	118	62	56	
Neoplasm histological type				0.514
Hepatocellular carcinoma	327	188	139	
Hepatocholangiocarcinoma	7	3	4	
Fibrolamellar carcinoma	3	1	2	
Neoplasm histological grade				0.373
G1	48	29	19	
G2	160	84	76	
G3	113	70	43	
G4	11	7	4	
Unknown	5	2	3	

### The HCC Immunogram

According to a previous study, the steps of the cancer-immunity cycle are described by eight axes of the immunogram score (IGS) as follows: IGS1, T cell immunity; IGS2, tumor antigenicity; IGS3, priming and activation; IGS4, trafficking and infiltration; IGS5, recognition of tumor cells; IGS6, inhibitor cells; GS7, checkpoint expression; and IGS8, inhibitory molecules (6). The gene sets IGS1, IGS3, IGS4, IGS5, IGS6, IGS7, and IGS8 were used in a previous study (6). A Gene Set Variation Analysis (GSVA) was performed to assess the value of IGS using GSVA R packages. The tumor neoantigenicity value was downloaded from published TCGA data (https://gdc.cancer.gov/about-data/publications/panimmune) (11). Unsupervised clustering of the IGS was performed using K-means clustering with the R package (version 3.6.1), as described in previous studies (12, 13). K-means clustering is one of most commonly used unsupervised machine learning algorithms (13). The HCC immunograms were classified into two clusters. The two clusters of the immunograms in radar plots are shown as the median IGS.

### Immune-Related Gene Signature

The gene sets for cytolytic activity (granzyme-A and perforin-1), the IFN-γ signature, immunocostimulators, immunoinhibitors, chemokines, HLA I signature, and HLA II signature were described in a previous study (14, 15). The immune signatures were measured as the geometric mean of gene expression in log2 of TPM+1.

### Molecular Features

The tumor neoantigen burden, tumor mutation burden, CNV burden scores, and LOH scores were derived from published TCGA data (9). Somatic alterations in 10 oncogenic signaling pathways were analyzed as previously described (16). We grouped genes into known 10 canonical pathways that included the cell cycle, Hippo, Myc, Notch, Nrf2, PI3 kinase/Akt, RTK-RAS, TGFβ signaling, p53, and β-catenin/WNT, as previously described. The sample in which genes in specific pathways contained somatic mutations was designated as specific pathway altered. The sample in which all genes in specific pathways were wild type was designated as specific pathway unaltered. The difference in the cancer pathway alteration frequency between the two HCC immunogram clusters was assessed using Fisher's exact test (two-sided).

### Statistical Analysis

Data are presented as means and standard errors of the means (SEM). Group values were assessed using a normal distribution test. For normally distributed data, group means were compared using Student's *t*-test, and non-parametric tests were performed when the data were not normally distributed. *P* < 0.05 was defined as statistically significant. Two-sided Fisher's exact test was used to compare alteration frequencies between patients with HCC presenting cold and hot HCC immunograms. The log-rank test was performed to investigate associations between the HCC immunogram patterns and the DFS and OS. Statistical analyses were performed using SPSS version 22.0 statistical software and R version 3.6.1.

## Results

### HCC Cancer Immunogram and Prognosis

**Figure 2:**
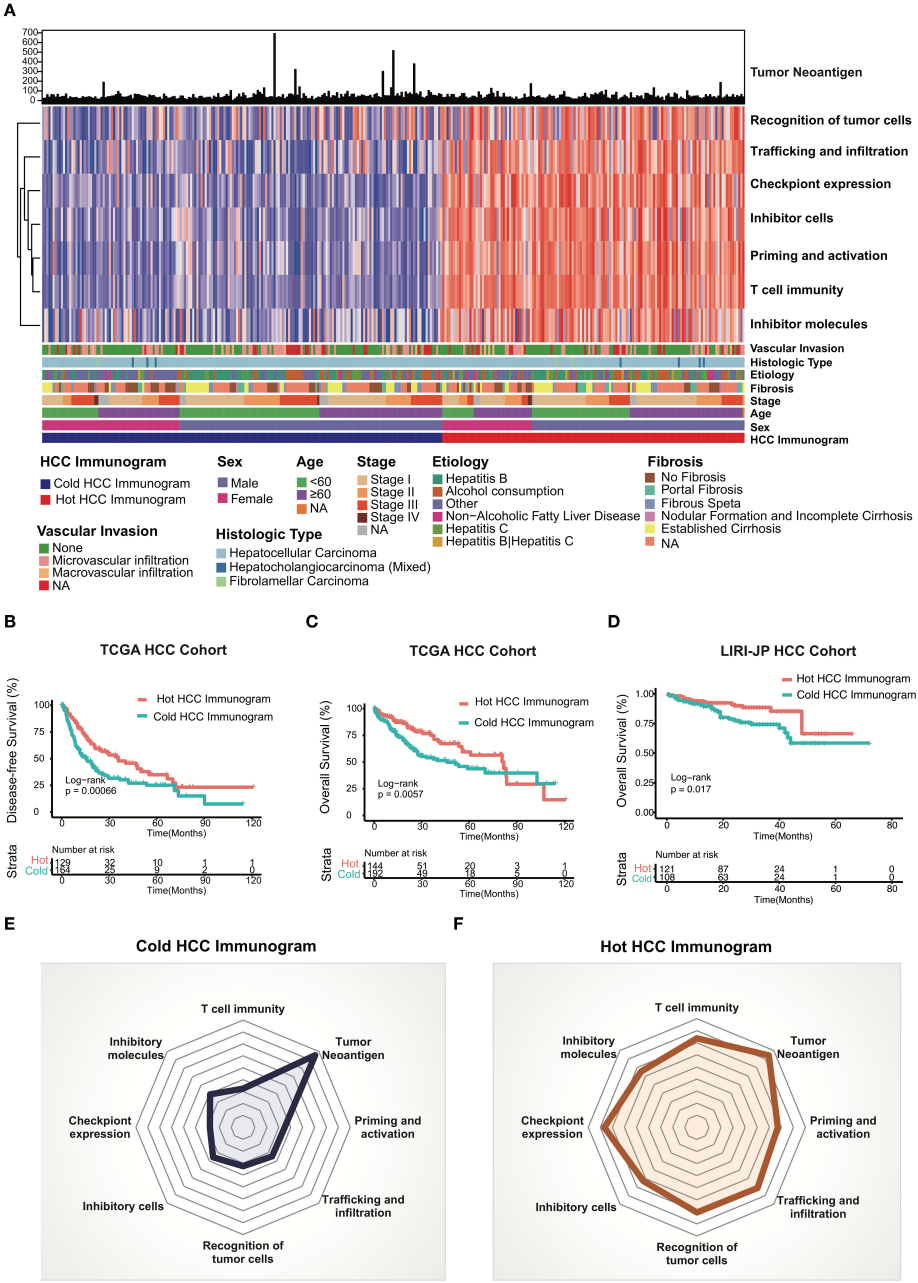
HCC cancer immunogram and prognosis. **(A)** Unsupervised clustering analysis of HCC immunograms based on eight axes of the IGS for 337 patients in the TCGA HCC cohort. The higher IGS cluster was termed the hot HCC immunogram, and the lower IGS cluster was termed the cold HCC immunogram. The clinical features, including age, sex, stage, fibrosis, etiology, histological type, and vascular invasion, are shown in patient annotations. **(B)** Kaplan–Meier curves for DFS of HCC patients in the TCGA cohort stratified into the two HCC immunogram clusters. The numbers of patients in the hot and cold immunogram clusters were 129 and 164, respectively. The log-rank test showed *P* = 0.00066. **(C)** Kaplan–Meier curves for OS of HCC patients in the TCGA cohort stratified into the two HCC immunogram clusters. The numbers of patients in the hot and cold immunogram clusters were 144 and 192, respectively. The log-rank test yielded *P* = 0.0057. **(D)** Kaplan–Meier curves for OS of HCC patients in the LIRI-JP HCC cohorts stratified into the two HCC immunogram clusters. The numbers of patients in the hot and cold immunogram clusters were 121 and 108, respectively. The log-rank test yielded *P* = 0.017. **(E,F)** The radar plot showed that the immunogram patterns of the two clusters were distinct. The axes of the radar chart were generated with the median IGS for the hot and cold immunogram clusters, respectively.

We adopted a cancer immunogram that visually illustrates the state of the cancer-immunity cycle to evaluate the antitumor response in patients with HCC. We explored the HCC immunogram of the TCGA cohort. Referring to a previous study, the steps of the cancer-immunity cycle are characterized by the following eight axes of the IGS: IGS1, T cell immunity; IGS2, tumor neoantigen burden; IGS3, priming and activation; IGS4, trafficking and infiltration; IGS5, recognition of tumor cells; IGS6, inhibitor cells; IGS7, checkpoint expression; and IGS8, inhibitor molecules. As shown in Figure 1, we collected the clinical data from the TCGA HCC cohort, WES, and RNA sequencing data. The landscape of the TCGA HCC cancer immunogram and the eight axes of the IGS are shown in Figure 2A. An unsupervised clustering analysis of the IGS was performed, and the HCC immunograms were separated into two clusters. The seven axes of the IGS of one cluster were significantly higher than the other cluster. The tumor neoantigen burden (IGS2) is not significantly changed between two clusters (Figure 2A and Supplementary Figure 1). The tumor neoantigen burden (IGS2) was not related to other axes of the immunogram, including T cell immunity (IGS1), priming and activation (IGS3), trafficking and infiltration (IGS4), recognition of tumor cells (IGS5), inhibitor cells (IGS6), checkpoint expression (IGS6), and inhibitor molecules (IGS8) (Supplementary Figure 2).

The cluster with higher immunogram scores was named the “hot immunogram,” and the cluster with lower immunogram scores was named the “cold immunogram.” Moreover, the two immunogram clusters of patients with HCC showed significant differences in DFS and OS (log-rank test, *P* < 0.01). Favorable OS and DFS were observed in patients with HCC and hot immunograms (Figures 2B,C). The radar plot showed that the immunogram patterns of the two clusters were distinct (Figures 2E,F). Furthermore, the relationship between clinical features and HCC immunogram patterns was investigated (Table 1). The immunogram patterns were not associated with clinical features, including age, gender, etiology, vascular invasion, fibrosis, stage, Child-Pugh classification grade, histological type, and neoplasm histological grade (Table 1).

The LIRI-JP HCC cohort was enrolled to test the HCC immunogram patterns and prognosis and to validate the prognostic value of the HCC immunograms. The clinical data, WES data, and RNA sequencing data were collected. HCC immunograms of patients with HCC in the LIRI-JP cohort were assessed and an unsupervised clustering analysis of the IGS was performed using the methods mentioned above. The results for the LIRI-JP cohort were similar to the TCGA cohort. The HCC immunograms were separated into two clusters termed the “hot immunogram” and “cold immunogram” according to the IGS. The OS of patients with HCC presenting the hot immunogram was longer than that of patients with the cold immunogram (log-rank test, *P* < 0.01, Figure 2D).

### The Immune Characteristics of Hot and Cold HCC Immunograms

**Figure 3:**
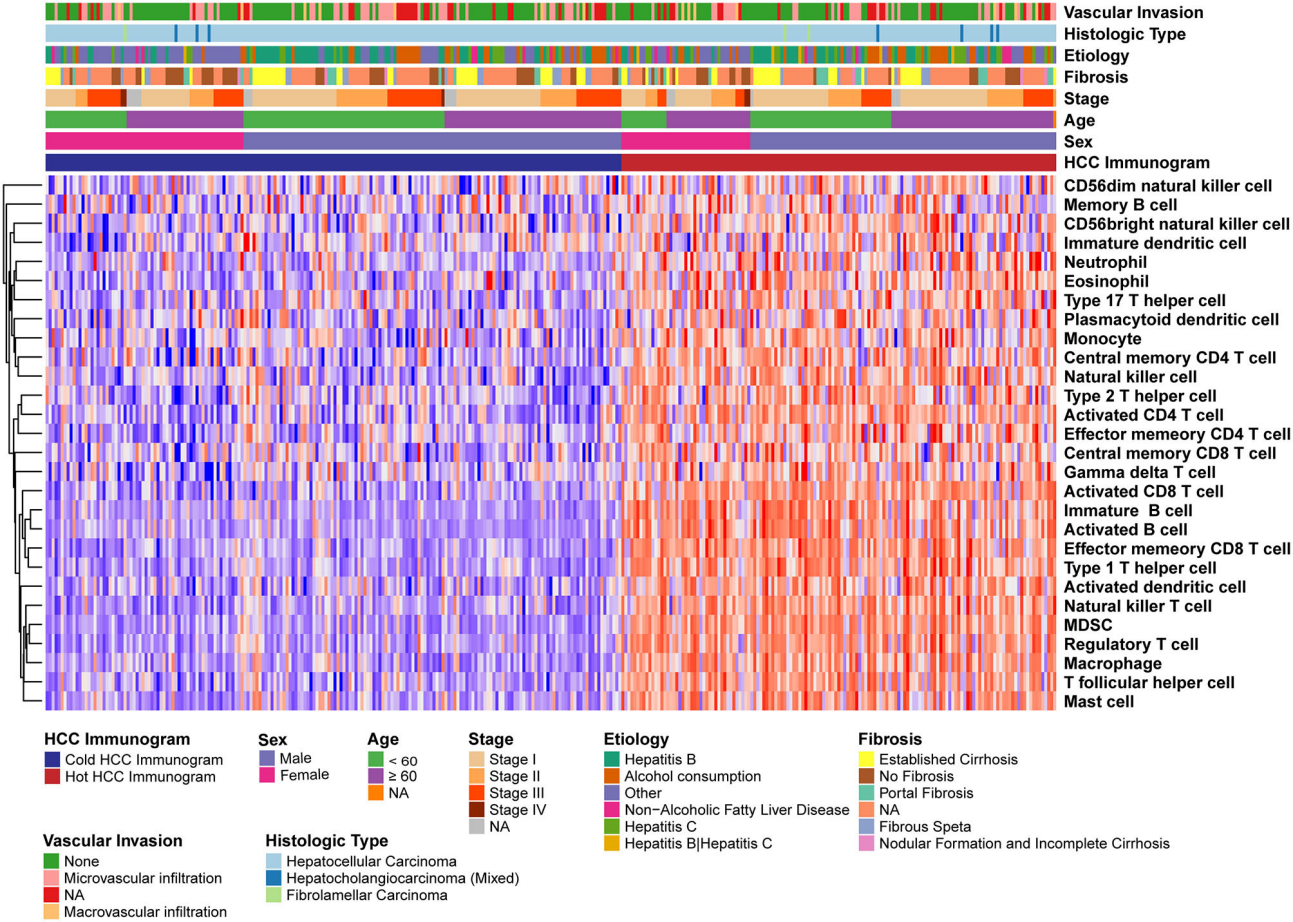
The heatmap of immune cell subsets that infiltrated the tumors of patients in the hot and cold HCC immunogram groups. The relative abundance of 28 immune cell subsets that infiltrated the tumor was evaluated with the sample level gene set enrichment method (GSVA) from the tumor RNA-Seq data. The clinical features, including age, sex, stage, fibrosis, etiology, histological type, and vascular invasion, are shown in the patient annotations.

**Figure 4:**
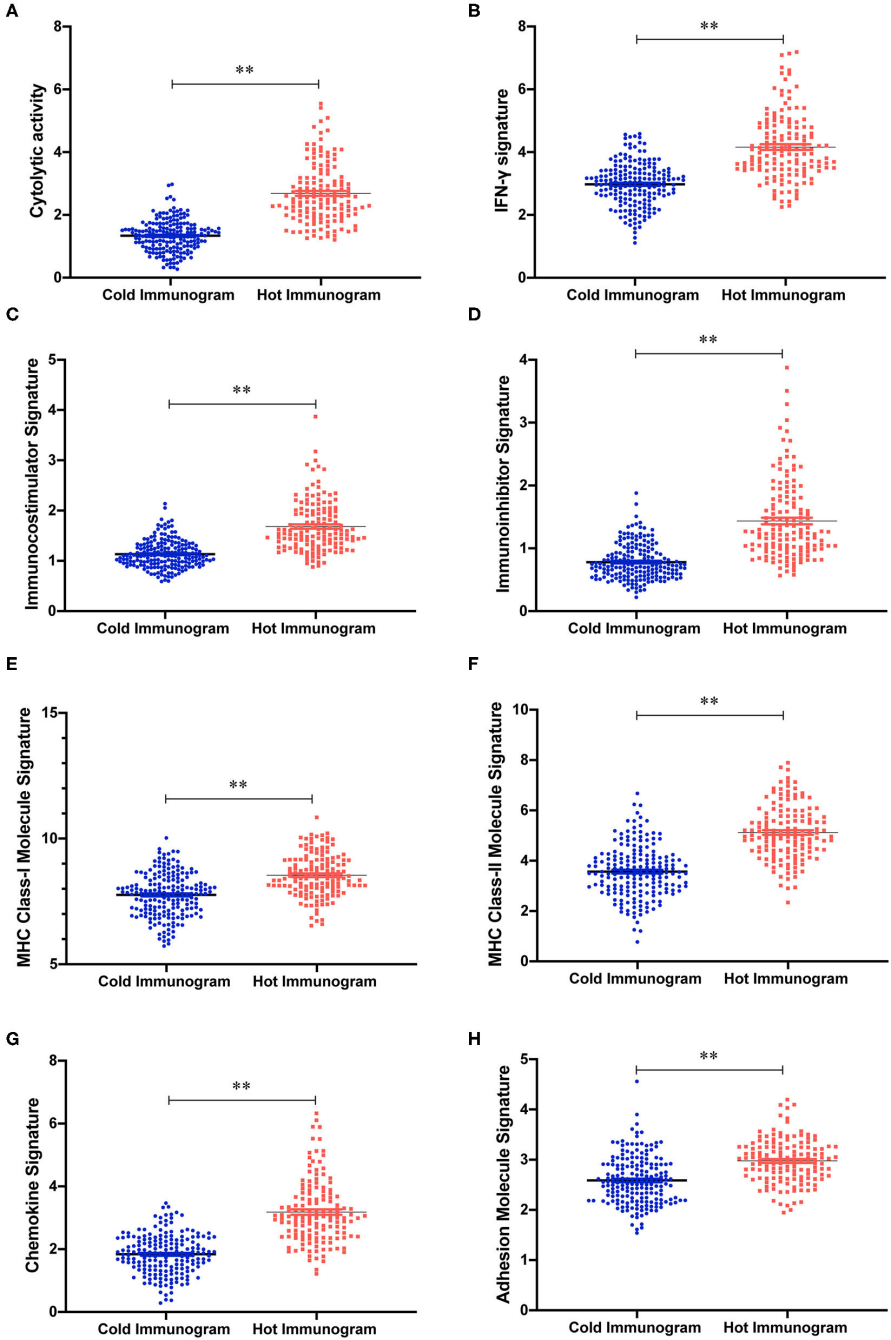
The immune signature strength of the hot and cold HCC immunogram groups. **(A–H)** The scatter plots showed higher levels of immune signatures, including cytolytic activity, cytolytic activity, IFN-γ signature, chemokine, immunoinhibitor, adhesion molecule, MHC I, MHC II, and non-class MHC, in hot HCC immunograms than in cold HCC immunograms. Group values were assessed using a normal distribution test. For normally distributed data, group means were compared using Student's *t*-test, and non-parametric tests were performed when the data were not normally distributed (*P < 0.05, **P < 0.01, and ns: not significant, *P* > 0.05).

The relative abundance of 28 immune cell subsets that infiltrate the tumor was evaluated using the single-sample Gene Set Enrichment Analysis (ssGSEA) method with the tumor RNA-Seq data. Immune cell infiltration was investigated in the two HCC immunogram clusters. As shown in Figure 3, greater numbers of 28 innate and adaptive immune cell subsets infiltrated the tumors of patients with hot immunograms than patients with cold immunograms. Furthermore, we observed higher levels of immune signatures, including cytolytic activity, IFN-γ signature, immunocostimulator, immunoinhibitor, chemokine, adhesion molecule, MHC I, MHC II, and non-class MHC, in hot HCC immunograms (*P* < 0.05, Figure 4).

### The Molecular Features of Hot and Cold HCC Immunograms

**Figure 5:**
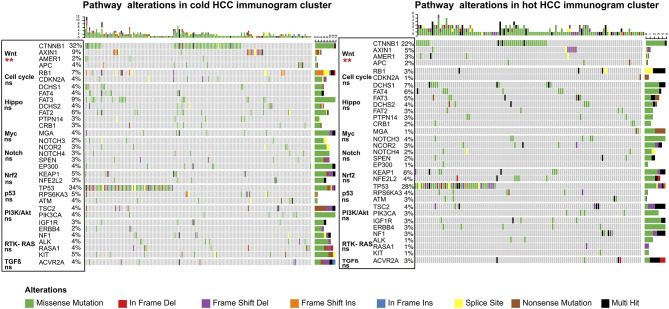
The alterations in cancer-related pathways identified in hot and cold HCC immunograms. The heatmap of the alterations in cancer-related pathways in hot and cold HCC immunograms. The difference in the frequency of alterations in cancer-related pathways between the two HCC immunogram clusters was assessed using Fisher's exact test (two-sided) (*P < 0.05, **P < 0.01, and ns: not significant, *P* > 0.05).

**Figure 6:**
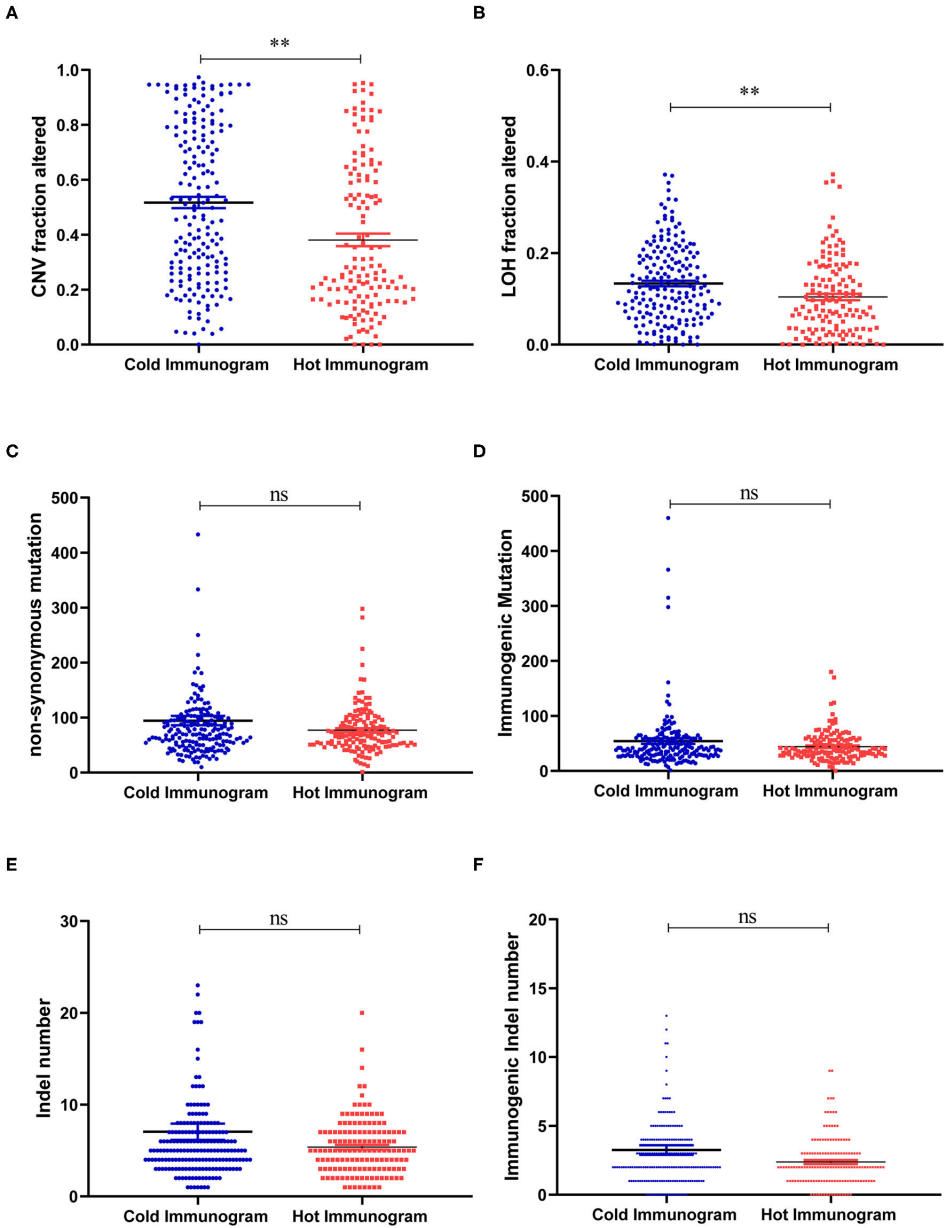
The molecular features of hot and cold HCC immunograms. **(A–F)** Scatter plots show the levels of molecular features, including CNV burden scores, LOH scores, non-synonymous mutations, immunogenic mutations, indel numbers, and immunogenic indel numbers in the hot and cold HCC immunogram clusters, respectively. Group values were assessed using a normal distribution test. For normally distributed data, means of two clusters were compared using Student's *t*-test, and non-parametric tests were performed when the data were not normally distributed (*P < 0.05, **P < 0.01, and ns: not significant, *P* > 0.05).

The driver gene mutations and signaling pathway alterations between the two HCC cancer immunogram clusters were investigated. The WNT pathway was altered and the CTNNB1 gene mutation frequency was higher in the cold HCC immunogram cluster than in the hot HCC immunogram cluster (two-sided Fisher's exact test, *P* < 0.05; Figure 5 and Table 2). The other signaling pathways, including the cell cycle, PI3K, P53, Notch, Myc, Hippo, Nrf2, and TGFβ pathways, were not significantly altered between the two HCC immunogram clusters (two-sided Fisher's exact test, *P* > 0.05; Figure 5 and Table 2). Moreover, higher CNV burden scores and LOH scores were observed in the cold HCC immunogram cluster than in the hot HCC immunogram cluster (*P* < 0.05, Figure 6). The other genetic variants, including non-synonymous mutations, immunogenic mutations, indel numbers, and immunogenic indel numbers, were not significantly altered (*P* > 0.05, Figure 6).

**Table 2:** HCC immunogram cluster and frequency of alterations in cancer-related pathways.

**Cancer pathway**	***N***	**Cold immunogram**	**Hot immunogram**	***P*-value (Fisher's Exact Test)**
**WNT pathway**				**0.008**
WNT altered	151	98	53	
WNT unaltered	184	92	92	
**TGFβ** **pathway**				0.493
TGFβ altered	20	13	7	
TGFβ unaltered	315	177	138	
**PI3K pathway**				0.788
PI3K altered	71	39	32	
PI3K unaltered	264	151	113	
**RTK/RAS pathway**				0.417
RTK/RAS altered	115	69	46	
RTK/RAS unaltered	220	121	99	
**Notch pathway**				0.432
Notch altered	77	47	30	
Notch unaltered	258	143	115	
**Myc pathway**				0.105
Myc altered	14	11	3	
Myc unaltered	321	179	142	
**Hippo pathway**				0.724
Hippo altered	108	63	45	
Hippo unaltered	227	127	100	
**Nrf2 pathway**				0.551
Nrf2 altered	28	14	14	
Nrf2 unaltered	307	176	131	
**Cell cycle pathway**				0.197
Cell cycle altered	44	29	15	
Cell cycle unaltered	291	161	130	
**P53 pathway**				0.139
P53 altered	127	79	48	
P53 unaltered	208	111	97	

*The bold values indicates P < 0.05*.

### The Immunogram Patterns of Molecular Features

**Figure 7:**
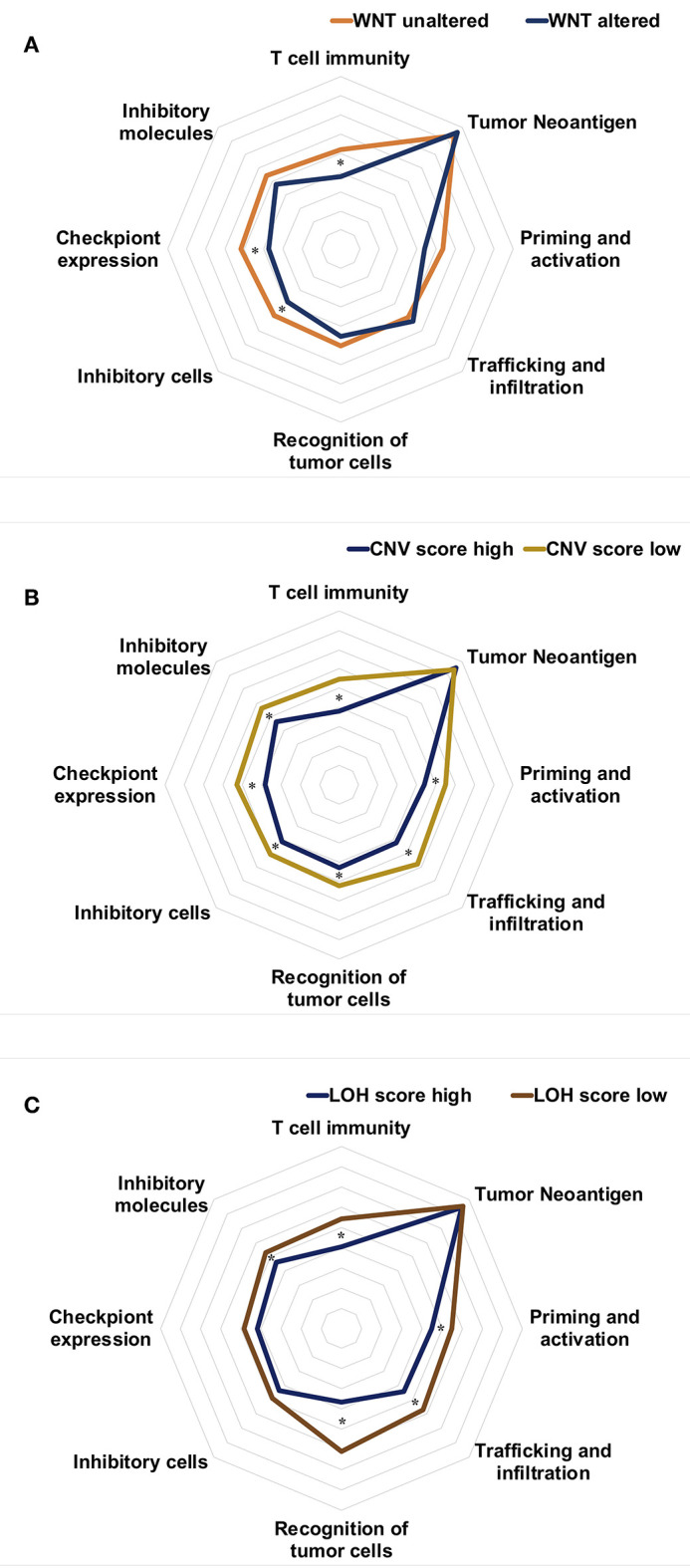
The immunogram patterns of molecular features. **(A–C)** Radar charts showing the immunogram patterns in tumors with and without Wnt-CTNNB1 alterations **(A)**, tumors with high and low CNV burdens **(B)**, and tumors with high and low LOH scores **(C)**. The median IGS are shown in the radar charts. Data were compared with non-parametric tests (*P < 0.05).

The main differences in molecular features between hot and cold immunograms were reflected in WNT-CTNNB1 alterations and CNV and LOH scores. We further investigated the immunogram patterns of tumors with different molecular features. As shown in Figure 7, the immunogram patterns were distinct for different molecular features of HCC tumors. The radar plot revealed higher IGS for T cell immunity, inhibitor cells, and checkpoint expression in tumors without Wnt-CTNNB1 alterations than in tumors in which Wnt-CTNNB1 was altered (*P* < 0.05, Figure 7A). Tumors with a high CNV burden were characterized by lower IGS for T cell immunity, priming and activation, trafficking and infiltration, recognition of tumor cells, inhibitor cells, checkpoint expression, and inhibitor molecules (*P* < 0.05, Figure 7B). Compared with tumors with a low LOH burden, tumors with a high LOH burden showed lower IGS for T cell immunity, priming and activation, trafficking and infiltration, and recognition of tumor cells and inhibitor molecules (*P* < 0.05, Figure 7C).

## Discussion

This study constructed an HCC immunogram of the cancer-immunity cycle to visually explore the anticancer immune responses of patients with HCC. The pattern of the HCC immunogram was categorized into two clusters, which were termed the hot immunogram and cold HCC immunogram. Favorable OS and DFS were observed for patients with HCC and hot immunograms. Moreover, the main difference in molecular features between hot and cold immunograms was reflected in WNT-CTNNB1 alterations and CNV and LOH scores. Meanwhile, the immunogram patterns were distinct for different molecular features of HCC tumors.

Based on emerging data, the anticancer immune response plays a vital role in cancer management (17–19). Previous studies reported an immunoscore based on an assessment of the numbers of CD3^+^ T cell and CD8^+^ T cells that infiltrated colon tumors (20). A recent study described three immunophenotypes based on CD8^+^ T cells, which included inflamed (CD8^+^ T cells infiltrated tumors, but were inhibited), immune excluded (CD8^+^ T cells accumulated, but had not efficiently infiltrated tumors), and immune desert (CD8^+^ T cells were absent from the tumor) (21). The immunophenotypes based on CD8^+^ T cells are helpful to understand the tumor microenvironment. Moreover, omics data from tumors provide additional information about the interaction of oncology and immunity. However, in clinical practice, clinicians must integrate data with multiple dimensions into a comprehensive visualization to assess the antitumor immune response and make appropriate clinical decisions for each patient. When the immunogram was utilized, the steps of the anticancer immune response of individual patients were described. In the present study, the different patterns of HCC immunograms were associated with different clinical outcomes. A significant benefit in terms of prognosis was observed in patients with HCC presenting hot immunograms. The results were validated in two independent HCC cohorts, including TCGA and LIRI-JP HCC cohorts. The antitumor response was likely activated in those patients, which reflected the higher IGS for T cell immunity, priming and activation, trafficking and infiltration, and recognition of tumor cells. In addition, greater numbers of infiltrated antitumor immune effector cells (activated CD8^+^ T cells and NK cells) and a stronger antitumor immune effector signature (cytolytic activity and IFN-γ) were associated with hot immunograms. Interestingly, the levels of immunoregulatory factors, including inhibitor cells, checkpoint expression, and inhibitor molecules, increased in patients with hot immunograms. We speculated that immunoregulatory factors exerted negative feedback on the activation of the antitumor immune response. The higher checkpoint expression associated with T cell-rich immunity and a strong immune effector signature (cytolytic activity and IFN-γ) may be related to the activation-exhaustion cascade in tumor-resident T cells (22).

The first step of the antitumor immune cycle is the release of tumor antigens and their capture by dendritic cells. Next, the dendritic cells present the captured antigens to T cells through MHCI and MHCII molecules, inducing the priming and activation of an effector T cell response against the cancer-specific antigens. However, the tumor neoantigen burden and tumor mutation burden of patients with HCC were not associated with T cell immunity, priming and activation, trafficking and infiltration, recognition of tumor cells, and antitumor immune effector signatures (cytolytic activity and IFN-γ). Our results were similar to the findings reported for patients with lung cancer (23). Based on these findings, progression from cancer neoantigen release to the T cell antitumor response involves multiple steps and complex mechanisms. As a single indicator, tumor neoantigens are unable to predict the antitumor immune response.

An understanding of the interaction between the tumor immune environment and molecular variations is vital to optimizing the immunotherapy strategy. In the present study, we investigated the alterations in 10 cancer-related pathways and molecular features between the two patterns of HCC immunograms. We observed a higher frequency of alterations in the WNT-CTNNB1 pathway in cold HCC immunogram pattern clusters. The immunogram patterns were distinct in tumors with and without WNT-CTNNB1 alterations. The radar plot showed higher IGS for T cell immunity, inhibitor cells, and checkpoint expression in tumors without Wnt-CTNNB1 alterations than in tumors with Wnt-CTNNB1 alterations. A clinical trial reported that patients with HCC carrying WNT/CTNNB1 mutations were resistant to immune checkpoint blockade (24). Our results may explain this immune resistance mechanism from the perspective of the HCC immunogram pattern. Moreover, our molecular analysis revealed higher CNV burden scores and LOH scores in the cold HCC immunogram cluster. The immunogram patterns of tumors with high CNV and LOH scores were characterized by lower IGS for T cell immunity, priming and activation, trafficking and infiltration, and recognition of tumor cells. A higher CNV and LOH burden in tumors correlated with immune escape and a poorer response to immunotherapy in previous studies (25, 26).

This study has several limitations. A further study should be designed to explore the clinical value of HCC immunograms in patient selection for personalized immunotherapy. From the perspective of theory, the individualized treatment strategies should be established based on the immunogram pattern for an assessment of the immune response of each patient.

In summary, a comprehensive understanding and assessment of the antitumor immune response is critical for medical decision making in cancer management. The present study used immunograms to provide visual antitumor immune response assessments for individual patients with HCC. Moreover, we illustrated the correlation between HCC immunograms and the molecular features of the tumor. This study may provide valuable resources for personalized HCC immunotherapy.

## Data Availability Statement

Publicly available datasets were analyzed in this study. This data can be found here: https://portal.gdc.cancer.gov/.

## Author Contributions

YH, HZ, and XW designed the study. YH and HS performed the data collection and analysis. YH explained the results and wrote the manuscript. All authors reviewed the manuscript.

## Conflict of Interest

HS was employed by the company Genecast Biotechnology Co., Ltd. The remaining authors declare that the research was conducted in the absence of any commercial or financial relationships that could be construed as a potential conflict of interest.

## Supplementary Material

Supplementary Figure 1 The boxplot shows the IGS of the hot and cold HCC immunogram clusters. Group values were assessed using a normal distribution test. For normally distributed data, mean values of the two clusters were compared using Student's *t*-test, and non-parametric tests were performed when the data were not normally distributed (^*^*P* < 0.05, ^**^*P* < 0.01, and ns: not significant, *P* > 0.05).

Supplementary Figure 2 The correlation of eight axes of the IGS in the HCC immunogram.

## Abbreviations

HCC: hepatocellular carcinoma
DFS: disease-free survival
OS: overall survival
CNV: copy number variant
LOH: loss of heterozygosity
TMB: tumor mutation burden
TCGA: The Cancer Genome Atlas.
