# Supplementary figures and images for: An Immunogram for an Individualized Assessment of the Antitumor Immune Response in Patients With Hepatocellular Carcinoma
Source: Front Oncol. 2020 Jul 31;10:1189. doi: 10.3389/fonc.2020.01189 (PMC7413104; doi:10.3389/fonc.2020.01189)

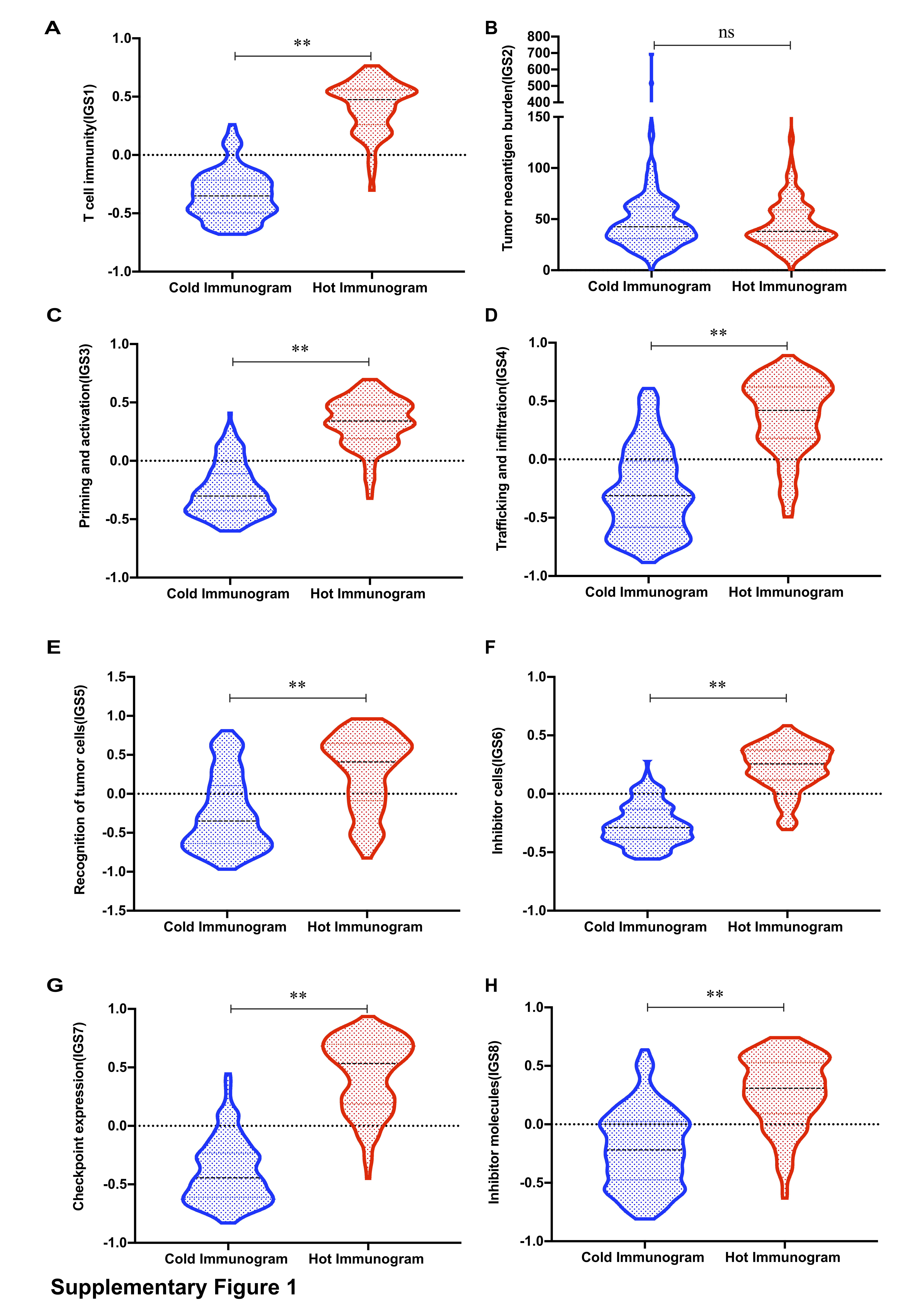

Supplement: Supplementary Figure 1 — The boxplot shows the IGS of the hot and cold HCC immunogram clusters. Group values were assessed using a normal distribution test. For normally distributed data, mean values of the two clusters were compared using Student's t-test, and non-parametric tests were performed when the data were not normally distributed (*P < 0.05, **P < 0.01, and ns: not significant, P > 0.05). [file Image_1.TIF]

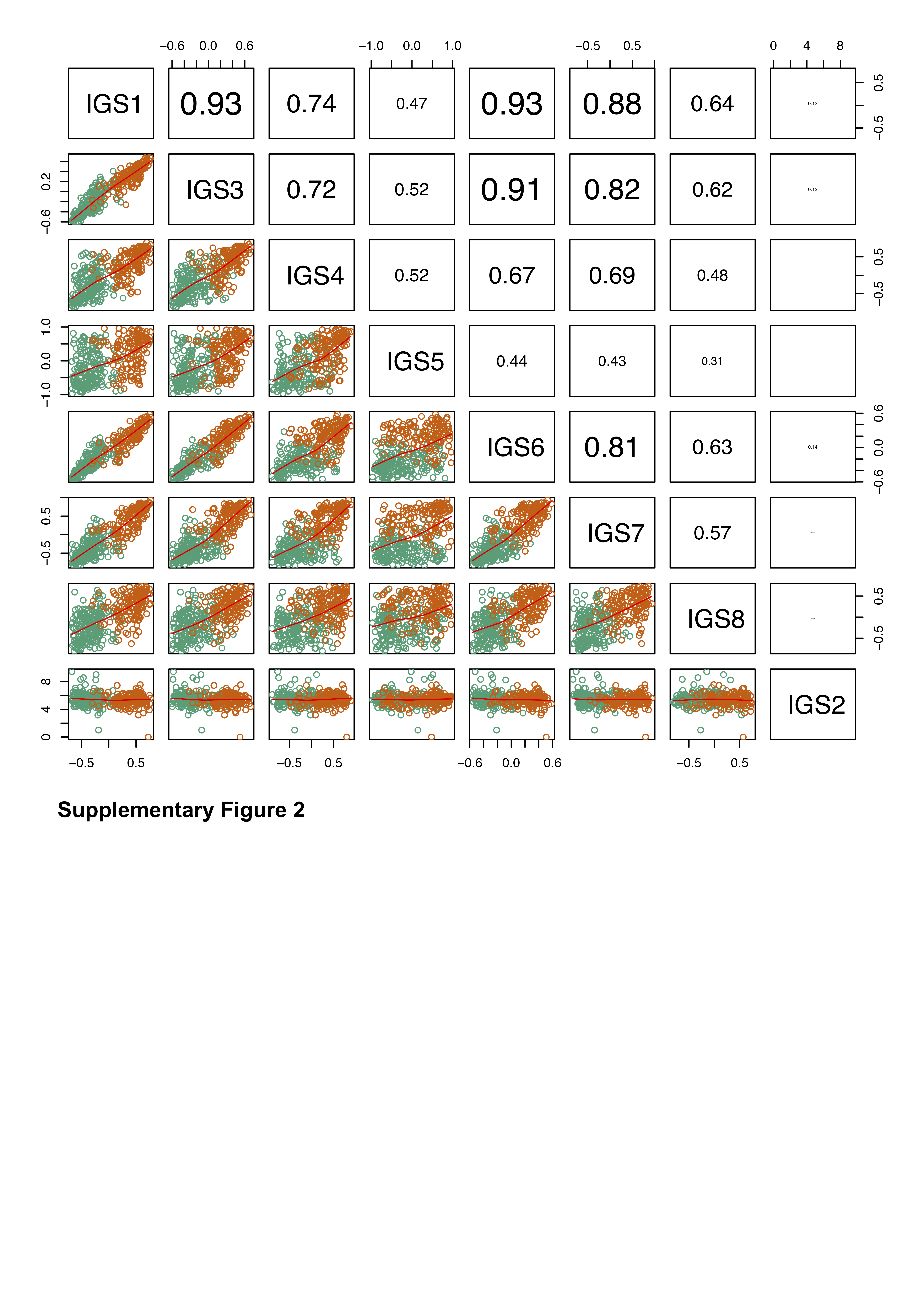

Supplement: Supplementary Figure 2 — The correlation of eight axes of the IGS in the HCC immunogram. [file Image_2.TIF]
